# Health Differences between Roma and Non-Roma in the Slovak Dialyzed Population

**DOI:** 10.3390/ijerph15020360

**Published:** 2018-02-18

**Authors:** Gabriel Kolvek, Zuzana Straussova, Maria Majernikova, Jaroslav Rosenberger, Jitse P. van Dijk

**Affiliations:** 1 Pediatric Department, Faculty of Medicine, Safarik University, 040 11 Kosice Slovak Republic; 2 Graduate School Kosice Institute for Society and Health, Faculty of Medicine, Safarik University, 040 11 Kosice, Slovak Republic; mamajern@gmail.com (M.M.); rosenberger.jaroslav@gmail.com (J.R.); j.p.van.dijk@umcg.nl (J.P.v.D.); 3 Nephrology and Dialysis Centre Fresenius Nitra, 949 01 Nitra Slovak Republic; zuzana.straussova@gmail.com; 4 Nephrology and Dialysis Centre Fresenius Kosice, 040 11Kosice, Slovak Republic; 5 Department of Community and Occupational Medicine, University Medical Center Groningen, University of Groningen, 9712 CP Groningen, The Netherlands

**Keywords:** Roma, health, dialysis, Slovakia

## Abstract

Background: Roma health has not been studied systematically. Thus far, it has been shown that Roma compared to non-Roma have a significantly higher likelihood of getting end-stage renal disease and that their chances for survival on dialysis are lower. Evidence is lacking regarding morbidity between Roma and non-Roma. The aim was to compare the health status of dialyzed Roma and non-Roma using the Charlson comorbidity index (CCI). All Slovak dialysis centers for adults were asked to fill in a questionaire with demographic and clinical data, including comorbidity. Cross-sectional analysis of 2082 patients with an average age of 63.8 ± 13.8 years was performed. Comorbidity was expressed as the CCI, and ethnic differences were calculated. Linear regression was performed to adjust for differences in gender and age in both ethnic groups. Roma represented 13.0% of the whole dialyzed population (n = 270). Comorbidity expressed as CCI was significantly lower in the Roma population (*p* < 0.001). After adjusting for gender and age, ethnicity failed to be associated with the CCI in the linear regression analysis (*p* = 0.965, variance of the model—adjusted R^2^ 38.6%). The health status of dialyzed Slovak Roma does not differ cross-sectionally when adjusted for age and gender from the health status of dialyzed non-Roma.

## 1. Introduction

Roma represent the largest minority in Slovakia [1]; however, their health has not been studied systematically. A higher prevalence of some chronic diseases, such as diseases of the cardiovascular system, diabetes mellitus, obesity as well as kidney disease, has been reported in the Roma minority [2,3,4,5,6,7]. So far, Roma compared to non-Roma have been shown to have a 2.4-times higher likelihood of getting end-stage renal disease (ESRD) [3] and that their chances for survival on dialysis are lower [8]. Sudzinova found a similar result in a survival study on cardiovascular disease [5]. While some part of the higher risk for ESRD might be explained by different occurrence of primary nephropathies [9], differences in comorbidity potentially explaining the shorter dialysis survival of Roma have never been identified. 

The Charlson comorbidity index (CCI) has been shown to be an accurate tool for assessing health status and survival prediction of diseased individuals; this tool has also been validated in dialyzed patients [10,11]. A study by Rattanasompattikul et al. [12] showed the CCI to be a robust and linear predictor of mortality, particularly in non-African Americans. The CCI has never been applied to (dialyzed) Roma. The aim of this study was to compare the health status of dialyzed Roma and non-Roma using the CCI. 

## 2. Materials and Methods

### 2.1. Patients and Procedure

All Slovak dialysis centers for adults were asked to fill in a questionaire with the following questions regarding patients: (a) demographic data—age, gender, ethnicity; (b) clinical data related to dialysis—date of dialysis start, primary nephropathy, dialysis modality, vascular access, weight, height, diabetes mellitus (DM); (c) complications and other diagnoses—complications of DM (other than renal failure), status after myocardial infarction and cerebrovascular accident, dementia, hemiplegia, chronic heart failure, chronic obstructive lung disease, solid tumor, leukemia, malignant lymphoma, ulcer disease, hepatopathy, AIDS, and connnective tissue disease. 

We performed this cross-sectional study in June and July 2017, with 39 out of 77 (response 50.6%) units participating in our study. We obtained data on 2082 patients (average age of 63.8 ± 13.8 years, 59.5% males, dialysis history of 4.6 ± 5.5 years). The only figure we had regarding the number of patients is that at the end of December 2016 there were 3453 prevalent dialyzed patients in the whole country (coverage of 60.2%) [13].

### 2.2. Measures

Body mass index (BMI) was calculated as the weight in kilograms divided by the square of the height in meters. 

To measure the comorbidity pattern of the patients we used the CCI; after scoring, the index was calculated [10].

We instructed that ethnicity be measured as follows: self-determination of ethnicity was compared to the determination judged by a physician, and in case of a mismatch the opinion of the head-nurse was decisive. This was necessary, as the self-determination of Roma in the last national census in 2011 showed a massive underestimation of the percentage of Roma (2.0%). Reliable estimates of Roma in Slovakia are several-fold higher [1].

### 2.3. Statistical Analyses

First, the Chi-squared test and Student’s *t*-test were used. Second, linear regression was performed to adjust for differences in gender and age in both ethnic groups. Analysis was considered statistically significant at the level of *p* < 0.05. The statistical software SPSS 23 (IBM Corp., Armonk, NY, USA) was used. 

## 3. Results

Out of 2082 patients from 39 dialysis units, 74% were being treated by hemodiafiltration (HDF), 23% hemodialysis (HD) and 3% were on peritoneal dialysis (PD). Seventy percent had an arteriovenous fistula (AVF), and 30% had a central venous catheter. The spectrum of diagnoses causing ESRD was as follows: diabetic nephropathy 34%, chronic tubulointerstitial nephritis 18%, chronic glomerulonephritis 13%, vascular nephrosclerosis 12%, others and unknown 10%, chronic pyelonephritis 6%, polycystic kidney disease 6% and vasculitis 1%. Ethnic differences in independent variables are shown in Table 1. 

Roma represented 13.0% of the whole dialyzed population (n = 270, the number of Roma in each dialysis unit varied from 0 to sometimes a very high percentage, up to 49%). Ethnic differences are presented in Table 2. Comorbidity expressed as the CCI was significantly lower in the Roma population (*p* < 0.001). After adjusting for gender and age, ethnicity failed to be associated with the CCI in the linear regression analysis (*p* = 0.965, variance of the model—adjusted R^2^ 38.6%; Table 3).

## 4. Discussion

We compared the comorbidity of Roma and non-Roma using the CCI in the Slovak dialyzed population; this is the first study in this field. We found that the CCI was significantly lower in the Roma population; however, after adjusting for the younger age of the Roma, the differences disappeared, showing that cross-sectionally, in patients surviving on dialysis, there are no significant differences in the health status of Roma and non-Roma. 

Roma ethnicity was shown to be an independent risk factor for mortality [8]. Similar to our results, the study of Gadalean et al. also showed a significantly younger age of dialyzed Roma and a shorter dialysis history. If the CCI is also a good predictor of mortality in the Roma ethnic group [12], then a higher CCI in Roma might be expected, which failed to be true in our sample. The explanation might be that patients with higher CCI are likely to die sooner and are therefore no longer present in our cross-sectional study, leading to the not-significant difference in CCI between ethnic groups. 

Ethnic differences in our study were also identified in the spectrum of diagnoses. “Others and unknown” were significantly more common in Roma (*p* = 0.001). Roma more frequently appear at dialysis units without previous follow-up by a nephrologist [14], which hampers precise diagnostics of their primary nephropathy and contributes to a higher risk of ESRD. Studies in other fields of medicine have similarly shown late referral of Roma [4,15], which adds to the higher morbidity of a specific disease in Roma. In our study, a comparison of the occurrence of diabetic nephropathy by ethnicity showed not-significant differences, and a similar proportion of patients with DM as well as its types separately (DM1 and DM2) were found not to differ in the mentioned subpopulations. The same holds true for complications of DM. This finding is in contrast with the higher prevalence of DM in the general Roma population in Slovakia as well as in the general population of inhabitants of India [6,16]. The Indian subcontinent is assumed to be the place of origin of the Roma. Our finding that the occurrence of DM does not differ between Roma and non-Roma can be explained by selection and survival bias of dialyzed patients. Polycystic kidney disease (PKD) was significantly less common among dialyzed Roma. This may be explained by the nature of this disease, which appears in family clusters. This seems to be in contrast to the situation in Hungary, where clusters of PKD were described as being more common among Roma with chronic kidney disease [17]—underlying an effect of a founder.

Significant differences by ethnicity were found in the modalities of dialysis; however, we may only speculate regarding the possible causes. The Slovak dialysis market is almost exclusively private, but it functions as a publicly organized system, accessible for every Slovak equally. The technical equipment and offered modalities vary dramatically among dialysis centers, however. In our questionnaire we did not ask for indications regarding any specific modality, so we can only guess as to the reason behind these differences. On the other hand, we found no significant ethnic differences in vascular access, which indirectly argues against discrimination. Ethnic comparisons of dialysis modalities are rarely described in the literature, i.e., in the U.S. renal data system (USRDS) no significant difference was found [18]. 

### 4.1. Strengths and Limitations

This is the first epidemiologic study in the Roma minority describing comorbidity using the CCI of the dialyzed population and representing more than half of the dialyzed Slovak population. However, some limitations should be mentioned. Although we did not have complete data on the whole dialyzed population, the participation of dialysis units from all parts of the country with a higher/lower share of Roma was only influenced by chance. This potential selection of the dialyzed population hampered any ethnic comparison of diabetes mellitus occurrence. Thus, this cross-sectional analysis, however national in its design, does not allow for causal interpretations. 

### 4.2. Implications

More attention should be paid to early stages of chronic kidney disease in Roma to prevent premature ESRD. Future studies should focus on ethnic differences in dialysis modalities to explain possible causes of these differences and on earlier mortality in the Roma patients compared to non-Roma. 

## 5. Conclusions

Cross-sectionally when adjusted for age and gender, no significant differences in health status expressed as the CCI of the dialyzed Roma and non-Roma in Slovakia were identified. However, the younger age of the dialyzed Roma and their shorter stay on dialysis suggests a higher mortality among Slovak dialyzed Roma. Longitudinal studies are needed to show mortality in (Slovak) Roma patients. 

## Figures and Tables

**Table 1:** Primary renal diseases causing end-stage renal disease (ESRD) by ethnicity.

Variable	Roma	Non-Roma	Difference
Diabetic nephropathy (%)	35.9	33.5	n.s.
Chronic tubulointerstitial nephritis (%)	14.4	18.7	n.s.
Chronic glomerulonephritis (%)	13.3	13.4	n.s.
Vascular nephrosclerosis (%)	11.1	12.4	n.s.
Others and unknown (%)	15.9	8.8	*p* = 0.001
Chronic pyelonephritis (%)	6.7	6.1	n.s.
Polycystic kidney disease (%)	1.9	6.0	*p* = 0.007
Vasculitis (%)	0.7	1.2	n.s.

n.s.—non-significant.

**Table 2:** Characteristics of the sample.

Variable	Roma	Non-Roma	Difference
Age (years)	55.0 ± 14.3	65.2 ± 13.3	*p* < 0.001
Dialysis history (years)	4.1 ± 4.3	4.7 ± 5.7	*p* = 0.034
Male gender (%)	53.0	61.0	*p* = 0.014
AVF (%)	68.9	69.7	*p* = 0.831
HD/HDF/PD (%)	38.7/60.2/1.1	20.3/75.8/3.9	*p* < 0.001
Weight (kg)	77.1 ± 22.4	76.1 ± 18.5	*p* = 0.414
Height (cm)	161.6 ± 11.5	167.3 ± 10.4	*p* < 0.001
BMI (kg/m^2^)	29.3 ± 7.7	27.1 ± 5.9	*p* < 0.001
CCI	5.92 ± 2.7	7.25 ± 2.9	*p* < 0.001

CCI: Charlson comorbidity index.

**Table 3:** Linear regression.

Model	B	95% CI	Difference
Ethnicity	−0.007	−0.321; 0.308	*p* = 0.965
Gender	0.303	0.091; 0.516	*p* = 0.005
Age	0.132	0.125; 0.140	*p* < 0.001

B—standardized coefficient; CI—confidence interval.
